# Conservative Management of Invasive Cervical Resorption Using Biodentine With Adjunctive Laser-Assisted Photodynamic and Photobiomodulation Therapy: A Case Report

**DOI:** 10.7759/cureus.107364

**Published:** 2026-04-19

**Authors:** Vandana Kumari, Savitha B Naik, Kiran Kumar Neelakantappa, Biji Brigit

**Affiliations:** 1 Department of Conservative Dentistry and Endodontics, Government Dental College and Research Institute, Bangalore, IND

**Keywords:** biodentine, invasive cervical root resorption, laser therapy, photobiomodulation, photodynamic therapy

## Abstract

Invasive cervical resorption (ICR) is an aggressive form of external root resorption that can cause significant structural damage if not detected early. This case report describes the conservative management of ICR using Biodentine (Septodont, Saint-Maur-des-Fossés, France) with adjunctive laser-assisted photodynamic therapy (PDT) and photobiomodulation therapy (PBM) to enhance disinfection and promote healing.

A 53-year-old female patient presented with a fractured maxillary central incisor and was diagnosed with Heithersay Class III ICR. Endodontic treatment was initiated, followed by surgical exposure of the resorptive defect. Granulation tissue was removed using an erbium-doped yttrium aluminum garnet (Er:YAG) laser (2,940 nm). Antimicrobial PDT was performed using 0.01% methylene blue activated with a 660 nm diode laser. The defect was restored with Biodentine. Postoperative PBM using an 810 nm diode laser was applied after suturing to enhance soft tissue healing.

Postoperative evaluation demonstrated satisfactory soft tissue healing at the surgical site without signs of inflammation or infection. Radiographic examination revealed restoration of the cervical defect with Biodentine and preservation of the surrounding periodontal structures. This multimodal laser-assisted approach may represent a predictable and minimally invasive strategy for the management of ICR.

## Introduction

Invasive cervical resorption (ICR) is an aggressive form of external root resorption characterized by progressive destruction of cervical dentin below the epithelial attachment. The lesion often remains asymptomatic in its early stages and may present clinically as a pinkish discoloration of the crown, while radiographically appearing as an irregular cervical radiolucency [1]. Predisposing factors include trauma, orthodontic treatment, intracoronal bleaching, and periodontal therapy [2,3]. Heithersay classified ICR into four categories based on the extent of dentinal invasion, which continues to guide treatment planning and prognosis assessment [2].

The primary objective in ICR management is complete removal of resorptive tissue, effective disinfection, and restoration with a biocompatible material that ensures an adequate seal and promotes repair [1]. Although mineral trioxide aggregate (MTA) has demonstrated favorable biological properties, its handling characteristics and prolonged setting time present clinical limitations.

Biodentine (Septodont, Saint-Maur-des-Fossés, France), a tricalcium silicate-based dentin substitute, has emerged as a promising alternative due to its improved handling, shorter setting time, and bioactive properties [4]. It exhibits mechanical characteristics comparable to natural dentin and stimulates mineralization through the release of growth factors such as TGF-β1 [4,5], making it suitable for restoration of cervical resorptive defects.

Microbial contamination within resorptive lacunae may compromise treatment outcomes. Adjunctive antimicrobial strategies such as photodynamic therapy (PDT) have demonstrated significant bacterial reduction in endodontic infections, particularly in areas inaccessible to mechanical instrumentation [6,7]. PDT involves activation of a photosensitizer by light of a specific wavelength, resulting in the production of reactive oxygen species that induce microbial cell death.

In addition to disinfection, modulation of postoperative healing may enhance clinical outcomes. Photobiomodulation therapy (PBM) stimulates mitochondrial activity, increases adenosine triphosphate (ATP) production, promotes angiogenesis, and reduces inflammation [8]. Low-level laser therapy has been shown to accelerate wound healing and improve tissue repair following dental surgical procedures. de Freitas and Hamblin reported that photobiomodulation enhances mitochondrial activity and ATP production, thereby promoting cellular proliferation and tissue repair [8]. Similarly, Gopal et al. [9] demonstrated in a systematic review that low-level laser therapy significantly improved soft tissue healing and reduced postoperative inflammation in oral surgical wounds.

Although Biodentine, PDT, and PBM have individually demonstrated satisfactory healing responses in restorative and endodontic procedures, evidence regarding their combined application in the conservative management of ICR remains limited. Therefore, this case report describes the use of Biodentine with adjunctive laser-assisted PDT and PBM in the management of ICR and evaluates its clinical outcome.

## Case presentation

**Figure 1:**
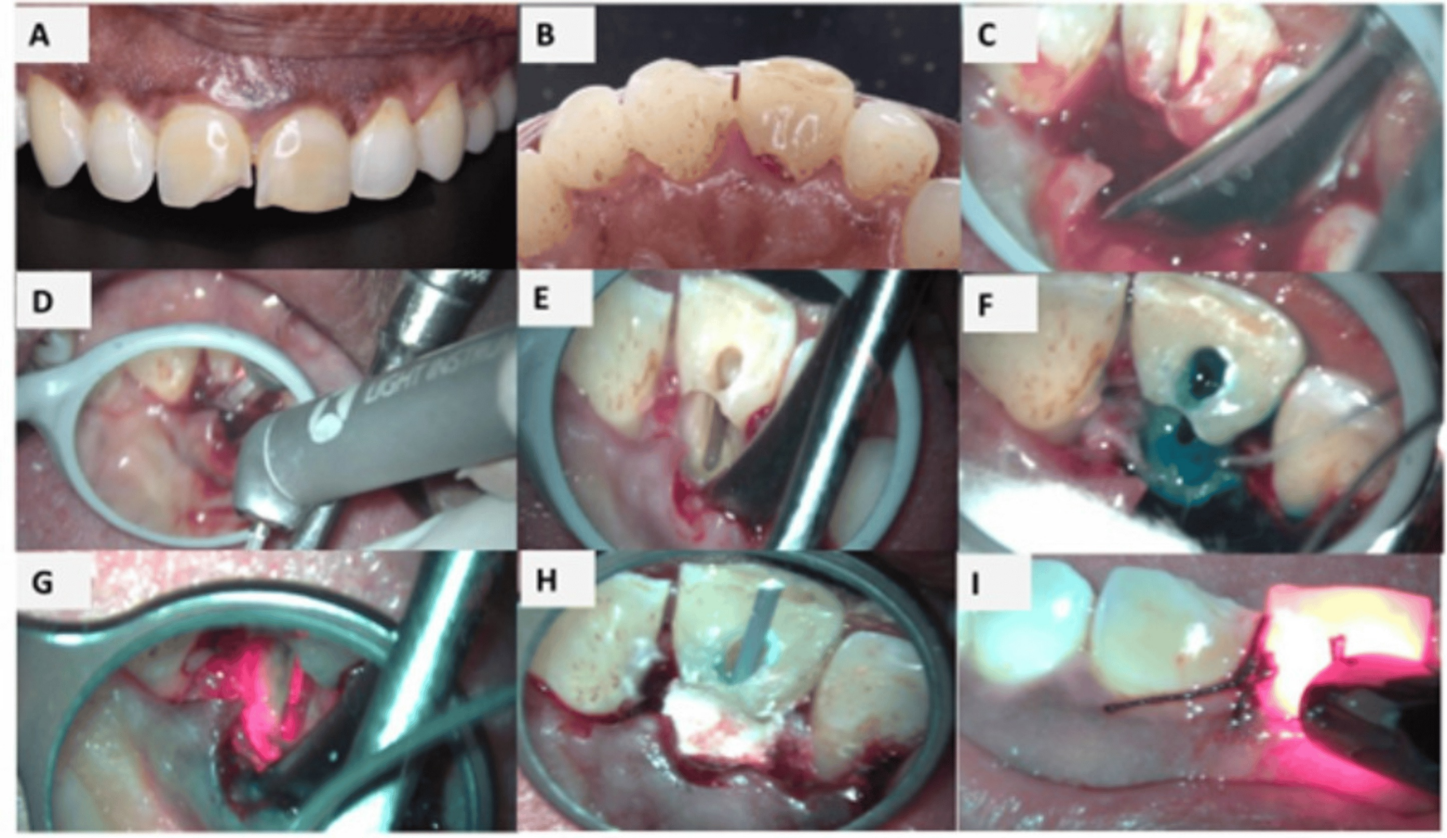
(A) Clinical image at first visit, (B) palatal image at first visit, (C) flap reflection during second visit for surgical management, (D) resection of necrotic dentin with LiteTouch™ Er:YAG Dental Laser, (E) complete extent of the resorptive surface, (F) pre-irradiation with methylene blue dye, (G) diode laser irradiation to perform photodynamic therapy, (H) Biodentine placed over the resorbed area to repair the defect, and (I) photobiomodulation with 810 nm diode laser

A 53-year-old female patient presented with the chief complaint of a fractured maxillary central incisor (Figure 1A). The patient reported a history of dental trauma approximately 10 years prior, for which no treatment had been sought. Clinical examination revealed an uncomplicated crown fracture involving enamel and dentin with respect to tooth #11. Periodontal probing around tooth #11 revealed a probing depth of 2-3 mm with no evidence of periodontal pocketing or bleeding on probing. On gentle probing, a distinct catch was detected on the palatal aspect, suggestive of underlying structural irregularity in the cervical region (Figure 1B). The tooth was asymptomatic; however, it exhibited no tenderness on percussion. Pulp vitality testing, including electric pulp testing and thermal (heat) testing, elicited no response, indicating pulpal necrosis.

**Figure 2:**
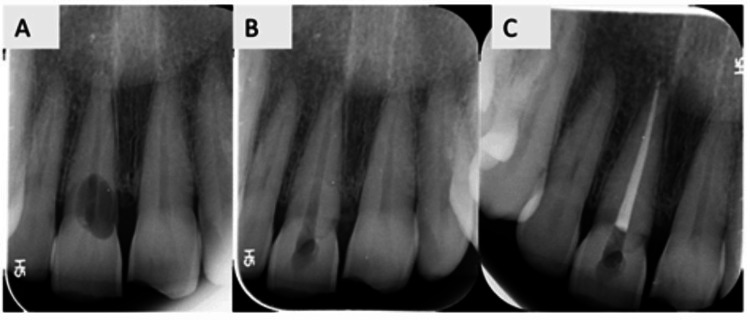
(A) Preoperative IOPAR, (B) postoperative radiograph after one week of surgical repair, and (C) IOPAR after root canal obturation IOPAR: intraoral periapical radiograph

Upon radiographic assessment, the intraoral periapical radiograph of the maxillary anterior region revealed a well-defined, irregular radiolucent lesion located in the cervical third of tooth #11 (Figure 2A). The radiolucency appeared asymmetrical and extended into the dentin, with a mottled internal pattern suggestive of resorptive activity. No evident periapical radiolucency was noted. The periodontal ligament space and lamina dura appeared largely preserved apically. The lesion was classified according to the Heithersay two-dimensional (2D) classification (1999) as class III (indicating a deeper invasion of dentin, extending into the coronal third of the root).

Treatment

Consent to Participate

Written informed consent was obtained from the patient for participation and for publication of clinical details and images. Following informed consent, endodontic treatment was initiated under local anesthesia using 2% lignocaine hydrochloride with 1:100,000 adrenaline, and the tooth was isolated with a rubber dam. Access cavity preparation was performed, and working length was established using an electronic apex locator (Root ZX Mini, J. Morita, Kyoto, Japan) followed by radiographic verification. A glide path was first negotiated with a #10 K-file, after which canal shaping was carried out using a rotary nickel-titanium system (ProTaper Gold, Dentsply Sirona, Charlotte, NC, USA). Coronal enlargement was achieved with S1 and S2 files, and apical preparation was completed up to size F3. Throughout instrumentation, copious irrigation was performed using normal saline, 17% ethylenediaminetetraacetic acid (EDTA), and 2.5% sodium hypochlorite to ensure adequate debridement and smear layer removal.

Calcium hydroxide paste (RC Cal, Prime Dental, Thane, India) was placed as an intracanal medicament and maintained for 14 days to enhance microbial control. The access cavity was temporarily sealed with Cavit™ G (3M, St. Paul, MN, USA), and the patient was scheduled for review after two weeks. As the resorptive lesion had established communication with the root canal system, a surgical intervention was planned to facilitate direct visualization and repair of the defect. Prior to surgery, the access cavity was temporarily sealed using a size 80, 0.02 taper gutta-percha cone to prevent ingress of debris while ensuring that the canal space remained unobstructed by permanent filling material. Subsequently, under local anesthesia, a full-thickness palatal mucosal flap was carefully elevated to expose the resorptive area under magnification 2.5x with Prima DNT dental microscope (Labomed, Fremont, CA, USA). Upon flap reflection, granulomatous tissue was evident along the palatal aspect of the tooth, extending coronally to the cervical third. The resorptive tissue was meticulously debrided using a surgical curette, and adequate hemostasis was subsequently achieved prior to further management. An erbium-doped yttrium aluminum garnet (Er:YAG) laser (LiteTouch™ Er:YAG Dental Laser, Light Instruments Ltd., Yokneam, Israel) equipped with a chisel tip was utilized for precise ablation and controlled removal of the defect while preserving the surrounding sound dentin and minimizing thermal damage (Figures 1D, 1E).

Laser parameters

Laser irradiation was performed using a LiteTouch™ Er:YAG Dental Laser system operating at a wavelength of 2,940 nm. A chisel tip was used in contact mode for laser application. The laser was set at an energy output of 100 mJ per pulse with a frequency of 10 Hz, delivering an average power of 1.0 W. The irradiation was carried out in short pulse (SP) mode. Continuous water spray cooling was used throughout the procedure to prevent thermal damage to the dentinal surface. The laser tip was applied in a light brushing contact with a gentle sweeping motion along the canal walls to ensure uniform irradiation of the dentin surface.

PDT with methylene blue using diode laser at 660 nm

Antimicrobial PDT was performed by introducing 0.01% methylene blue into the defect and allowing a 60 s pre-irradiation time to ensure adequate photosensitizer penetration (Figure 1F). The site was then irradiated using a 660 nm diode laser (SOLASE, Lazon Medical Laser Co., Ltd., China) at 100 mW in continuous wave mode for 60 s through a 200 µm fiber tip, thereby activating the dye and promoting singlet oxygen-mediated antimicrobial effects (Figure 1G). Following completion of PDT, the defect was thoroughly irrigated with sterile saline to remove residual photosensitizer. The operative field was then carefully isolated and dried to ensure optimal conditions for the placement of Biodentine to repair the resorptive defect.

Repair of resorptive defect

Biodentine was prepared by mixing the powder and liquid components according to the manufacturer’s instructions and was carefully adapted into the resorptive defect, conforming to the natural tooth contours. An initial setting period of approximately 12 minutes was allowed before proceeding further (Figure 1H). After initial setting of Biodentine, the flap was repositioned and secured using single interrupted sutures with silk suture 6-0 PERMA-HAND™ Silk Suture (Ethicon, Inc., Somerville, NJ, USA).

Photobiomodulation with diode laser

**Figure 3:**
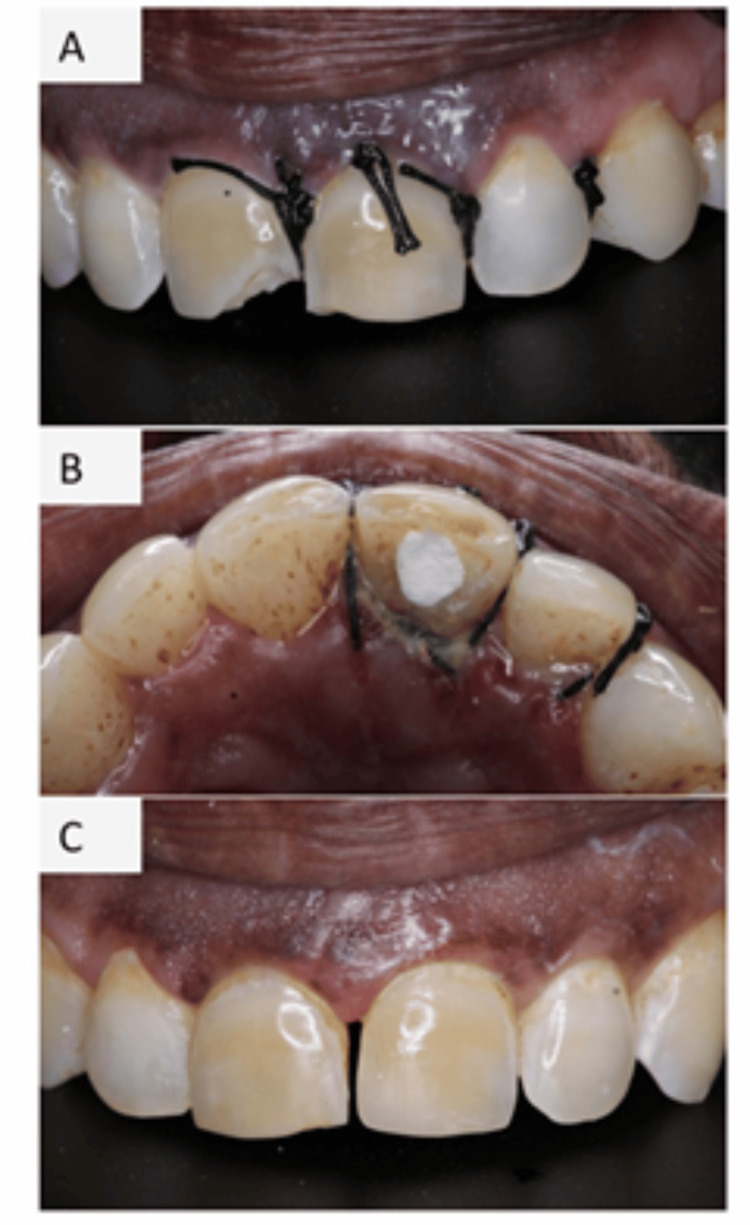
(A, B) Postoperative clinical images after one week of surgery, (C) composite restoration completed for central incisors after one month

After placement of sutures, PBM was delivered using an 810 nm diode laser (SOLASE) in non-contact mode at an output power of 100 mW for 60 s per point. Irradiation was performed using a standard 300 µm fiber tip in a defocused, sweeping motion over the surgical site to enhance angiogenesis, modulate inflammation, and promote postoperative soft tissue healing (Figure 1I). One week after surgery, the patient was recalled for clinical evaluation and suture removal. Root canal treatment was then completed using the lateral condensation technique. Postoperative clinical photographs (Figures 3A, 3B) and radiographs (Figures 2B, 2C) were obtained. Composite restoration for both the central incisors was completed after one month (Figure 3C).

Clinical outcome evaluation included assessment of pain or discomfort, periodontal probing depth, tooth mobility, and soft tissue healing at the surgical site. Radiographic evaluation was performed to assess restoration of the cervical defect, integrity of the periodontal ligament space, and absence of periapical pathology.

One-Week Follow-Up

One week after surgery, the patient was recalled for clinical evaluation and suture removal (Figures 2B, 3A). The surgical site showed satisfactory healing with no signs of infection, swelling, or postoperative complications. The patient reported minimal discomfort and satisfactory oral function. Oral hygiene instructions were reinforced, and the treated tooth remained asymptomatic with no tenderness on percussion or palpation.

One-Month Follow-Up

At the one-month follow-up visit, the patient remained asymptomatic, and clinical examination revealed satisfactory soft tissue healing with healthy gingival architecture around the treated site (Figure 3C). The tooth was functional with no evidence of mobility or tenderness on percussion or palpation. Radiographic evaluation demonstrated satisfactory healing of the periapical region with no signs of pathology (Figure 2C).

## Discussion

ICR presents a significant clinical challenge due to its often asymptomatic progression and potential for extensive structural destruction before diagnosis [1,2]. Early identification and complete removal of resorptive tissue are critical for arresting lesion progression and preserving the affected tooth. In the present case, a history of trauma likely acted as a predisposing factor, as traumatic injury has been consistently associated with the initiation of cervical resorptive processes [2,3]. Radiographic findings demonstrated an irregular cervical radiolucency with preservation of the root canal outline, consistent with the external origin of the lesion [1]. Accurate diagnosis and assessment of lesion extent are fundamental for selecting a conservative treatment strategy.

Root canal therapy was initiated prior to surgical repair due to pulpal necrosis and communication of the resorptive defect with the canal space. Elimination of intracanal infection is essential to prevent persistent inflammation and treatment failure [10]. Placement of calcium hydroxide as an intracanal medicament provided additional antimicrobial action and contributed to an alkaline environment unfavorable to osteoclastic activity [11].

Surgical exposure allowed direct visualization and complete debridement of the defect. The use of an Er:YAG laser (2,940 nm) for the removal of residual granulation tissue offered several advantages over conventional mechanical curettage. Owing to its high absorption in water, the Er:YAG wavelength enables precise microexplosive ablation with minimal thermal diffusion to adjacent hard tissues [12,13]. Studies have demonstrated its effectiveness in removing inflamed tissue while preserving surrounding dentin and promoting a clean surgical field [13]. Furthermore, laser-assisted debridement may contribute to surface decontamination and improved healing dynamics.

Adjunctive antimicrobial PDT was performed using 0.01% methylene blue activated by a 660 nm diode laser. PDT generates reactive oxygen species, including singlet oxygen, which exert cytotoxic effects on microorganisms without inducing bacterial resistance [6]. Previous investigations have shown significant bacterial reduction when PDT is used as an adjunct to conventional chemomechanical or surgical debridement, particularly in anatomically complex areas where instrumentation may be limited [6,7]. In resorptive defects with irregular lacunae, this adjunctive disinfection approach may enhance microbial control and improve prognosis.

Following disinfection, the defect was restored with Biodentine, a calcium silicate-based bioactive material. Biodentine exhibits favorable mechanical properties, excellent sealing ability, and bioactivity through calcium ion release [4]. Laurent et al. demonstrated that Biodentine stimulates the release of growth factors such as TGF-β1, promoting mineralization and reparative dentin formation [5]. Compared with MTA, Biodentine offers improved handling characteristics and a shorter setting time, which are advantageous in surgical settings [4]. Its dentin-like modulus of elasticity may also contribute to structural reinforcement of cervical defects.

Postoperative PBM was administered using an 810 nm diode laser to enhance tissue healing. PBM exerts its biological effects through mitochondrial chromophore activation, particularly cytochrome c oxidase, resulting in increased ATP production, modulation of inflammatory mediators, and stimulation of angiogenesis [8]. Clinical evidence suggests that PBM accelerates soft tissue repair and reduces postoperative inflammation and discomfort following oral surgical procedures [8,9]. In the present case, PBM was employed to optimize wound healing and support periodontal tissue regeneration around the repaired defect. At the one-week follow-up, the surgical site exhibited satisfactory soft tissue healing without signs of infection or inflammation. The patient reported minimal postoperative discomfort, indicating an uneventful healing period.

The integration of Er:YAG laser-assisted debridement, antimicrobial PDT, bioactive restoration with Biodentine, and postoperative PBM represents a biologically driven and minimally invasive approach to ICR management. While individual modalities have been well documented in dental literature, reports describing their combined application in ICR remain limited. The favorable clinical and radiographic outcome observed in this case suggests that synergistic use of laser-based disinfection and biostimulation with bioactive restorative materials may enhance treatment predictability and long-term stability. The patient was recalled after one week for suture removal and clinical evaluation.

Conventional management of ICR typically involves mechanical curettage of the resorptive tissue followed by restoration using materials such as MTA or glass ionomer cement. While these methods have demonstrated clinical success, mechanical instrumentation alone may not always achieve complete removal of resorptive tissue within irregular lacunae. In the present case, the use of an Er:YAG laser allowed precise ablation of necrotic dentin while preserving surrounding dentin. Additionally, adjunctive antimicrobial PDT may enhance microbial reduction in areas that are difficult to access with conventional instrumentation.

Postoperative radiographs confirmed restoration of the cervical defect. Following completion of root canal treatment, the tooth was restored with composite resin. The clinical and radiographic findings supporting the outcome of the treatment are illustrated in Figures 1-3, which demonstrate the initial presentation of the lesion, surgical management of the resorptive defect, placement of Biodentine, and postoperative healing. Further clinical studies with larger sample sizes and long-term follow-up are required to substantiate the benefits of combined laser-assisted protocols in the management of ICR.

## Conclusions

The present case demonstrates that ICR can be successfully managed using a conservative, biologically driven approach integrating surgical debridement, Er:YAG laser-assisted granulation tissue removal, adjunctive antimicrobial PDT, restoration with a bioactive calcium silicate material (Biodentine), and postoperative PBM. The combined use of laser-based disinfection and biostimulation with a bioactive restorative material facilitated effective microbial control, structural repair, and favorable soft tissue healing. Within the limitations of a single case report, this multimodal laser-assisted protocol appears to enhance clinical predictability and may represent a promising minimally invasive strategy for the management of ICR. Further controlled clinical studies with long-term follow-up are warranted to validate these findings.
